# Metabolomic Analysis of Environmental Biomarkers Reveals Markers of Mate Preference in Female Giant Pandas

**DOI:** 10.3390/ani15192873

**Published:** 2025-09-30

**Authors:** Yongyou Feng, Jing Ke, Xiangming Huang, Maohua Wang, Mingxi Li, Jingchao Lan, Kongju Wu, Linjie Wang

**Affiliations:** 1 The Conservation of Endangered Wildlife Key Laboratory of Sichuan Province, Chengdu Research Base of Giant Panda Breeding, Chengdu 610081, China; pandahxm@sina.cn (X.H.); lmxtr@hotmail.com (M.L.); lanjingchao@panda.org.cn (J.L.); 15228901362@163.com (K.W.); 2 College of Animal Science and Technology, Sichuan Agricultural University, Chengdu 611130, China; kejing@stu.sicau.edu.cn (J.K.); 2024302134@stu.sicau.edu.cn (M.W.)

**Keywords:** giant panda, mate preference, environmental biomarkers, metabolites

## Abstract

**Simple Summary:**

Giant pandas are conservation reliant. Natural mating is more successful than artificial insemination. Mate preference is a critical factor in the natural reproduction of giant pandas, but it is poorly understood. Given the importance of improving the reproductive efficiency of the giant panda, this study focused on the physiological and metabolic changes in female giant pandas during mate preference trials conducted at estrus. In this study, we collected environmental biomarkers from 19 female pandas during mate preference trials with 3 males and performed metabolomics analysis. We identified several differential metabolites, including prostaglandin G2, prostaglandin E2, and estrone, which are potentially associated with female mate preference. In addition, through KEGG pathway enrichment analysis, we found that female mate preference was related to steroid hormone biosynthesis, phenylalanine metabolism, and tropane, piperidine and pyridine alkaloid biosynthesis. These results reveal potential physiological markers related to female mate preference, providing insights for formulating effective conservation and breeding strategies for the giant panda.

**Abstract:**

The giant panda (*Ailuropoda melanoleuca*) is a vulnerable animal in China, and it is crucial to improve the reproduction efficiency of the giant panda. Mate preference is an important part of natural mating. We hypothesized that AGS metabolites differ according to their mate preference. In this study, we determined estrus-associated hormone levels in the urine of 19 female giant pandas. After confirming estrus via hormone levels and behavioral observation, we collected environmental biomarkers for metabolomics analysis. A total of 19 samples were divided to two groups according to the mating preference of female giant pandas. Metabolomics analysis by LC-MS/MS showed that a total of 115 differentially expressed metabolites were identified, including 97 upregulated metabolites and 18 downregulated metabolites. We found that prostaglandin B2, palmitoylcarnitine, prostaglandin G2, and estrone may be the potential markers of female mate preference. Pathway enrichment analysis showed that steroid hormone biosynthesis, phenylalanine metabolism, and tropane, piperidine, and pyridine alkaloid biosynthesis were the top three pathways. These results revealed the physiological changes in female giant pandas during mate preference trials, providing a perspective for understanding their chemical communication system reliant on anal gland secretions and improving the success rate of natural mating of giant pandas.

## 1. Introduction

The giant panda is a solitary large mammal that relies on a highly complex chemical communication system to support social interaction and reproduction [1,2]. Giant pandas rub anogenital gland secretions onto tree trunks and rocks as information carriers to display individual status and social behaviors [3,4]. However, the specific informational content conveyed by these environmental biomarkers remains poorly understood.

Nowadays, assisted reproduction technology has been widely applied in livestock breeding. But for giant pandas, it does not work as effectively as for other animals. The male giant pandas need to be anesthetized prior to semen collection, with appropriate electrical stimulation then being administered, which poses safety risks to them [5]. Although semen can be cryopreserved at −196 °C for a long time [6], the cryopreservation process itself exerts negative impacts on DNA structural stability [7]. Giant pandas exhibit an estrous cycle with interval of more than 12 months, while maintaining a remarkably narrow fertility window of approximately 48 h during each cycle [8], which makes it more challenging to determine the optimal timing for semen deposition. In addition, an analysis of 300 fertilization events reveals that the probability of offspring production through natural mating is 60.7%, while the fertility rate of artificial insemination is only 19.5% [9]. Consequently, it is feasible to improve the reproductive rate of giant pandas by improving the success rate of natural mating.

Mate preference is an important part of natural mating. Understanding the physiological changes in the female giant panda mate preference trial can further improve the success rate of natural mating. The total successful intromission is 72.00% when female giant pandas mate with their preferred males, compared to only 31.25% with none-preferred males. Natural mating with preferred mates can enhance females’ reproductive success [10]. Giant panda pairs exhibiting mutual preference achieve a 90% cub production rate while pairs with reciprocal aversion demonstrate complete reproductive failure (0% cub production) [11]. Only those giant pandas exhibiting high-frequency courtship behaviors by both partners succeed in mating and producing cubs, demonstrating that mate preference critically determines mating success [12].

Because of the solitary behavior of giant pandas, olfactory communication is the primary mechanism for evaluating reproductive status in conspecifics [13]. Both urine and anogenital gland secretions (AGS) serve as chemical signals, effectively communicating key biological information including individual identity, sex, reproductive status, age, and social dominance rank [14]. The previous study has revealed that the volatile constituents in AGS of giant pandas consist of complex mixtures of 30 to 50 compounds, including fatty acids, aldehydes, ketones, aliphatic ethers, amines, aromatic compounds, alcohols, steroids, and squalene [2]. Another study has shown that a total of 120 chemical compounds are identified in the AGS and environmental biomarker samples of giant pandas, including fatty acid esters, alkanes, alcohols, sterols, and over half of them are unique to females [15]. Furthermore, the relative abundance of many compounds varies significantly with the season, while the concentration of volatile compounds is notably higher during the mating season [16]. The evidence above indicates that the chemical constituents in giant panda AGS may be closely implicated in conveying breeding-related information.

However, it remains unclear whether metabolites in AGS are related to female giant panda mate preference. Therefore, we hypothesized that AGS metabolites differ between preferred and non-preferred group females. LC-MS/MS analysis was conducted to identify metabolites in the environmental biomarkers derived from the AGS of female giant pandas potentially associated with mate preference. In our study, a total of 115 differential metabolites were identified between the preferred and non-preferred groups. Pathway enrichment analysis revealed these differential metabolites were primarily enriched in the steroid hormone biosynthesis pathway. Our study provides a novel insight into the evaluation of female giant panda mate preference through their environmental biomarkers.

## 2. Materials and Methods

### 2.1. Animals

During the estrous season, a total of 19 female giant pandas (*Ailuropoda melanoleuca*) in natural estrus and 3 potential male mates from the Chengdu Research Base of Giant Panda Breeding (Chengdu, Sichuan Province, China) were included in this study from January to March 2024. Females with prior normal estrous cycles and age-matched, reproductively competent males were selected. Bamboo culms and leaves constituted over 90% of the captive giant panda diet, supplemented by fruits, vegetables, and corn/wheat concentrates. After estrus identification, every female giant panda was exposed to potential male mates, respectively, and mating attempts were initiated after a 3-day period.

### 2.2. Estrus Identification by Behavioral Observation

During the estrous season, female giant pandas exhibit heightened vigilance and restlessness, accompanied by distinct behavioral alterations. They engaged in repetitive pacing within their activity areas, reduced food intake, and displayed apparent agitation. Additionally, urination, scent-marking, and olfactory investigation behaviors occurred more frequently to augment chemical communication. Then, they initiated proximity to males and adopted mating-readiness postures. Characteristic actions included backing against objects while pressing the hindquarters and elevating the pelvis when the tail base is gently stimulated with a bamboo rod. Acoustic signals intensified and featured frequent bleat-like vocalizations and avian-like calls.

### 2.3. Estrus Identification by ELISA

To identify estrus in female giant pandas, we employed ELISA to detect estrus-associated hormones levels in urine. Specifically, urine samples were collected daily from female individuals during the estrous season. The collected urine samples were centrifuged at 3000 rpm for 10 min, and the supernatant was collected for hormone assay. The samples were analyzed for estrogen conjugate (EC) levels and creatinine (Cr). We assessed estrogen metabolism status by measuring the levels of estrogen conjugates in urine. Specifically, we selected one of the predominant glucuronide-conjugated forms Estrone-3-glucuronide (E1G) as a representative biomarker. Due to the stability of urinary Cr concentration, we utilized it to standardize measurements against dilution effects. The concentration of Estrone-3-glucuronide and creatinine was measured using Estrone-3-Glucuronide ELISA Kits (Arbor Assays, Ann Arbor, MI, USA) and Creatinine Urinary ELISA Kits (Arbor Assays, Ann Arbor, MI, USA), respectively, following the manufacturer’s instructions. The ratio in female giant pandas during peak estrus period typically ranges from 80 to 150 ng/mg [17]. Based on an EC/Cr ratio combined with behavioral observations, we determined the optimal timing to place females and males in adjacent enclosures for trials.

### 2.4. Collection and Classification of Environmental Biomarkers Derived from Anogenital Gland Secretions (AGS)

Upon confirming estrus via ELISA and behavioral observation, every female giant panda was housed in an enclosure adjacent to a male giant panda. If a female panda exhibited extreme rejection behaviors (e.g., emitting threat calls or physical rejection) toward a male giant panda, she was introduced to two alternative males. Environmental biomarkers from females that exhibited strong rejection behaviors toward all 3 males were directly classified into the non-preference group. The absence of such behaviors triggered mating attempts on the final day of the trial. Female giant pandas that failed mating with male giant pandas were classified into the none-preferred group as well, while individuals that succeeded in mating attempts were classified into the preferred group.

When a female giant panda did not exhibit the behavior toward a particular male individual, or she demonstrated the behavior toward all 3 male individuals, we began to collect environmental biomarkers daily throughout their trial period. We used cotton swabs to collect the environmental biomarkers from the ground within her enclosure twice daily at 10:00 a.m. and 10:00 p.m., continuing for 3 consecutive days from the onset of estrus. All 6 swabs used to collect environmental biomarker samples from the same giant panda were combined into a single 50 mL centrifuge tube as a composite sample. All collected samples were immediately stored in centrifuge tubes and flash-frozen in liquid nitrogen, then stored at −20 °C. A total of 19 composite samples were collected, consisting of 10 from the preferred group and 9 from the non-preferred group.

### 2.5. LC-MS/MS

Chromatographic separation was performed on a Thermo Vanquish UPLC system (Thermo Fisher Scientific, Waltham, MA, USA) equipped with an ACQUITY UPLC^®^ HSS T3 column (2.1 × 100 mm, 1.8 μm; Waters, Milford, MA, USA). Mass spectrometry analysis was conducted using a Thermo Orbitrap Exploris 120 instrument (Thermo Fisher Scientific, Waltham, MA, USA) with an ESI source. We aliquoted equal amounts from each sample to form a quality control (QC) pool, which was used to correct for biases in the analysis of mixed samples and instrumental errors. For LC-ESI (+)-MS analysis, the mobile phases consisted of (B2) 0.1% formic acid in acetonitrile (*v*/*v*) and (A2) 0.1% formic acid in water (*v*/*v*). Separation was conducted under the following gradient: 0~1 min, 10% B2; 1~5 min, 10%~98% B2; 5~6.5 min, 98% B2; 6.5~6.6 min, 98%~10% B2; 6.6~8 min, 10% B2. For LC-ESI (−)-MS analysis, the analytes were carried out with (B3) acetonitrile and (A3) ammonium formate (5 mM). Separation was conducted under the following gradient: 0~1 min, 10% B3; 1~5 min, 10%~98% B3; 5~6.5 min, 98% B3; 6.5~6.6 min, 98%~10% B3; 6.6~8 min, 10% B3. The mass spectrometry parameters were as follows: sheath gas pressure, 40 arb; aux gas flow, 10 arb; spray voltage, 3.50 kV and −2.50 kV for ESI(+) and ESI(−), respectively; and capillary temperature, 325. Mass spectrometric analysis was performed using an Orbitrap-based instrument. Full MS scans were acquired at a resolving power of 60,000 (FWHM) over the mass range m/z 100–1000. Data-dependent MS/MS acquisition was triggered for the top 4 most intense ions per cycle, fragmented with a normalized collision energy of 30%, and analyzed at a resolving power of 15,000 (FWHM). A dynamic exclusion function was applied with an automatic setting.

After obtaining the base peak chromatogram, peak detection, peak filtering, and peak alignment were performed using the R XCMS software package (version 3.12.0). Metabolites with an effective proportion above 70% were filtered, and the data were normalized by raw peak area values/total peak area values. Molecular formula prediction was conducted by considering mass deviation and adduct ion information, followed by database matching.

### 2.6. Statistics Analysis

Differences in EC/Cr levels were analyzed using one-way ANOVA. Significance analysis was determined by Duncan’s multiple range tests (Graphpad prism 10). A supervised multivariate statistical analysis model (OPLS-DA) was applied to discriminate the groups by BioDeep (https://www.biodeep.cn, 18 May 2025). The statistical significance was determined by a two-tailed unpaired Student’s *t*-test. We employed FDR (False Discovery Rate) multiple-testing correction to adjust the t-values. Finally, we combined *p* value and VIP (OPLS-DA variable projection importance) to screen differential metabolites. The metabolites (*p* value < 0.05, VIP value > 1) were considered to be differential metabolites.

Upon mapping differential metabolites to KEGG pathways, functional enrichment analysis of metabolic pathways was performed using the hypergeometric distribution. Subsequently, topological analysis was conducted to evaluate the relative importance of metabolites within biochemical networks. The relative metabolites and associated KEGG pathways were presented by the KEGG Mapper tool (https://www.kegg.jp, 6 June 2025).

## 3. Results

### 3.1. Estrus-Associated Hormone Levels in the Urine of 19 Female Giant Pandas

**Figure 1:**
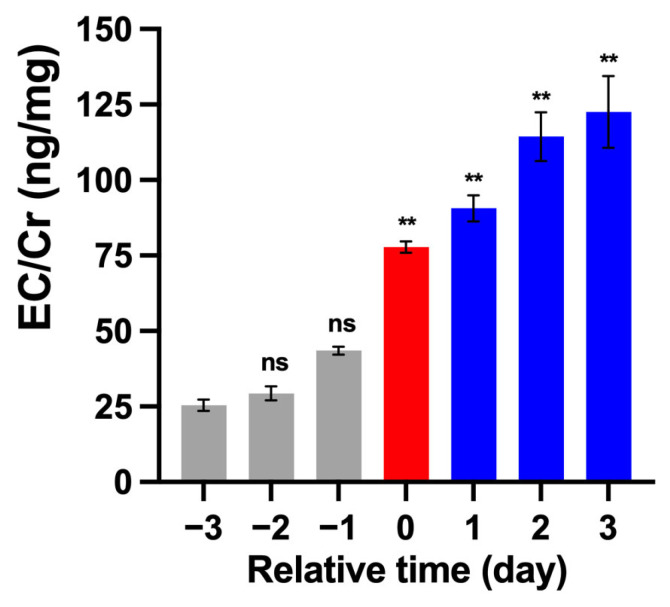
Urinary estrogen conjugates-to-creatinine ratio in female giant pandas before and after Day 0. Day 0 is defined as the start of estrus, and Day 1 to Day 3 constituted the trial period. “Relative time” refers to the day relative to Day 0. The values are presented as the mean ± SEM. Significant differences compared with Day −3 are indicated by asterisks: ** *p* < 0.01. “ns” means not significant.

Behavioral changes in female giant pandas were observed, followed by measurement of the urinary estrogen conjugates-to-creatinine ratio via ELISA in 19 individuals. This ratio was used to identify estrus onset, thereby establishing the optimal sampling window. An EC/Cr ratio exceeding 80 ng/mg can be used as a reliable indicator of a peak estrus period in female giant pandas. Starting from Day −3, EC/Cr levels exhibited a sustained increase. On Day 0, the value reached 77.86 ng/mg and showed a significant difference compared to Day −3 (*p* < 0.01). Moreover, levels remained above 80 ng/mg during the trial period and reached a peak at 122.57 ng/mg on Day 3. These results provide reliable evidence that these pandas entered their peak estrus period after Day 0 and maintained this status throughout the trial period, thereby providing a confirmed estrus timing for initiating sample collection from Day 1 (Figure 1).

### 3.2. Global Features of Metabolic Profiles of Two Groups Environmental Biomarkers from Female Giant Pandas

**Figure 2:**
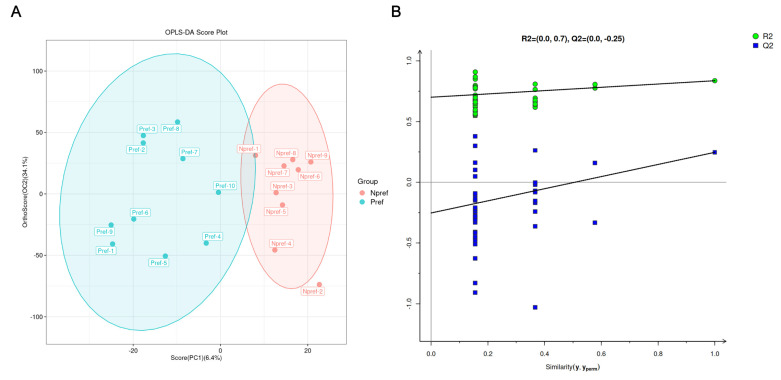
Global features of metabolic profiles of the two groups’ environmental biomarkers from female giant pandas. (**A**) The OPLS-DA analysis for discriminating against the metabolite profiles of giant panda environmental biomarkers of the Pref group (blue) and the Npref group (red), with each dot representing one sample from each group. (**B**) The OPLS-DA permutation test chart. Q^2^Y intercept ≤ 0 indicates that the structure is reliable and effective.

A total of 19 environmental biomarker samples were classified into the preferred group (Pref, n = 10) and the non-preferred (Npref, n = 9) group based on the female mating preference. To investigate the physiological changes in female giant pandas in mate preference trials, metabolomics analysis was performed to detect environmental biomarkers derived from the AGS of female giant pandas. We found that all samples fell within the quality control confidence intervals, and 80.1% of samples exhibited a relative standard deviation (RSD) ≤ 30%, indicating the overall high quality and reliability of the metabolomic dataset. Samples with RSD ≤ 30% were subjected to downstream analysis. Furthermore, the OPLS-DA model parameters of the Pref and the Npref group were R2X = 0.405, R2Y = 0.837, and Q2 = 0.247 (Figure 2A). The OPLS-DA permutation test charts illustrated the reliability and validity of the construction (*p* = 0.0396, Figure 2B). The Q2 value indicates a model with limited predictive ability, suggesting subtle global metabolic differences between groups. Therefore, our focus will shift to metabolites that are both statistically significant in univariate analysis and exhibit substantial fold changes, as these are more likely to represent true biological alterations.

### 3.3. Differential Metabolites Screening Between the Pref and Npref Group

**Figure 3:**
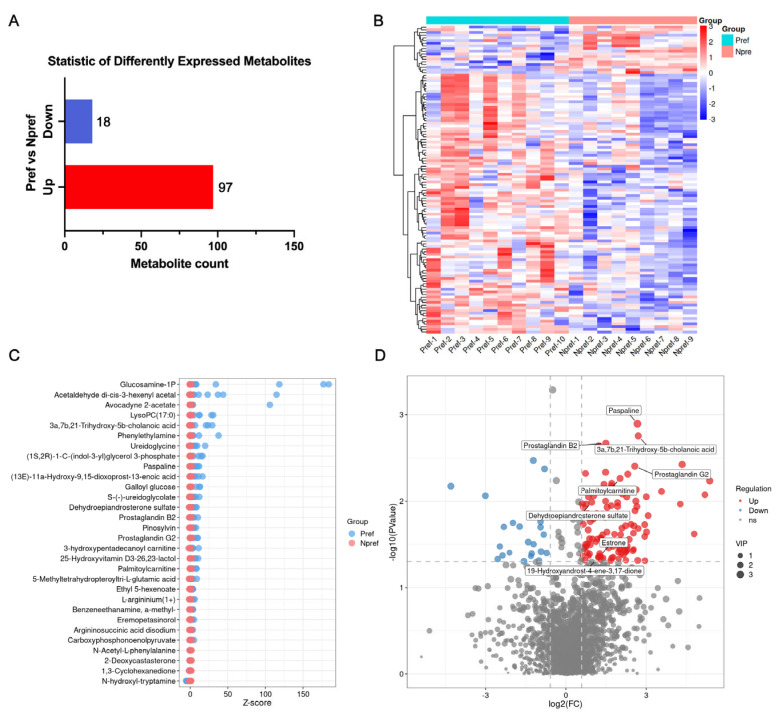
Differential metabolites screening between the Pref and Npref groups. (**A**) Statistic of differently expressed metabolites. (**B**) Clustering heatmap of differential metabolites. (**C**) Z-score distribution of differential metabolites. Horizontal position reflects relative abundance across samples. Right-shift in Pref group indicates upregulation. (**D**) The volcanic plot of all metabolites. Red dots represent significantly upregulated metabolites, and blue dots represent significantly downregulated metabolites.

After FDR multiple-testing correction was applied to all metabolites, no metabolites showed significant differences (q < 0.05). Therefore, we used *p* < 0.05 and VIP > 1 as the screening thresholds to obtain the list of differential metabolites. A total of 115 metabolites were significantly changed between the Pref and Npref group, including 97 upregulated and 18 downregulated metabolites (Figure 3A). The list of differential metabolites was presented in Table S1. The heatmap of differential metabolites based on their relative quantity was plotted (Figure 3B). Furthermore, we screened the top 30 differential metabolites ranked by -log10 (*p* value) and plotted the Z-score plot (Figure 3C). We found that glucosamine-1P, acetaldehyde di-cis-3-hexenyl acetal, and avocadyne 2-acetate were the top three metabolites with relative content in the samples of the Pref group. The volcanic plot of all metabolites between the Pref group and Npref group was depicted. Differential metabolites that could potentially be markers of mate preference for female giant pandas were annotated, including prostaglandin B2, palmitoylcarnitine, prostaglandin G2, estrone, and so on (Figure 3D). These metabolites are closely associated with reproductive functions and most likely represent potential markers in environmental biomarkers related to mate preference.

### 3.4. Pathway Enrichment Analysis of Differential Metabolites Between the Pref and Npref Groups

**Figure 4:**
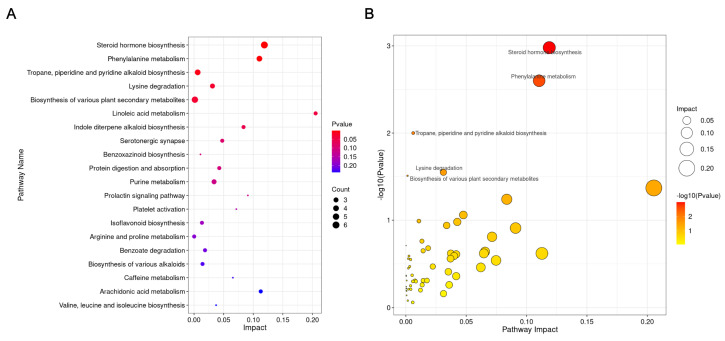
Pathway enrichment analysis of differential metabolites between the Pref and Npref groups. (**A**) The bubble map of pathways enriched by differential metabolites. (**B**) The scatter plot of metabolic pathway impact factors.

Differential metabolites identified between the Pref and Npref groups were subjected to pathway enrichment analysis. The KEGG pathway enrichment results for the top 20 metabolic pathways potentially associated with female giant panda mate preference are presented in the bubble plot (Figure 4A). The scatter plot depicting the impact factors of metabolic pathways highlights the top five significantly altered pathways (Figure 4B). The top five pathways were steroid hormone biosynthesis, phenylalanine metabolism, tropane, piperidine and pyridine alkaloid biosynthesis, lysine degradation, and biosynthesis of various plant secondary metabolites.

## 4. Discussion

As a first-class protected animal and national treasure in China, research on the reproductive efficiency and mechanisms of the giant panda is critically important [18,19]. The present study focused on the physiological features of female giant panda mate preferences, aiming to identify mate preference biomarkers. After employing 3 male and 19 female giant pandas for a mate preference trial, we collected environmental biomarkers derived from the AGS of female giant pandas. We employed metabolomic approaches to screen for differential metabolites between the preferred and non-preferred groups. There were 97 significantly upregulated differential metabolites in the preferred group compared to the non-preferred group. The top three relative contents were glucosamine-1P, acetaldehyde di-cis-3-hexenyl acetal, and avocadyne 2-acetate. It has been suggested that glucosamine plays a key role in development as a crucial precursor of the glycosylation reaction. Glucosamine-1P is considered a potential marker of reproductive functions in animals [20]. Therefore, we hypothesize that glucosamine-1P might be one of the metabolites associated with mate preference in the AGS of female giant pandas. However, acetaldehyde di-cis-3-hexenyl acetal and avocadyne 2-acetate are likely dietary-derived metabolites that may reflect differences in diet rather than exhibit strong associations with mate preference.

The significantly upregulated components that may serve as biomarkers for mate preference in female giant pandas were labeled in the metabolite volcano plot. Among them, prostaglandin G2 is involved in PGF2α biosynthesis and prostaglandin B2 is a degradation product of prostaglandin E2. It has been reported that PGF2α can initiate oestrous behavior and follicular development [21]. In addition, prostaglandin E2 is involved in ovulation, fertilization, and uterine repair in female mammals [22,23]. Palmitoylcarnitine, a well-known intermediate in fatty acid oxidation, can regulate the generation of progenitor cells in the maternal tissue [24]. Estrone, as a metabolic precursor form of estradiol, ensures basal hormone levels and can be applied to the estrus identification of animals [25]. In addition, 19-Hydroxyandrost-4-ene-3,17-dione, a precursor to estrogen synthesis, also plays an important role in steroid metabolism [26]. A previous study has shown that dehydroepiandrosterone sulfate not only plays an important role as an intermediate in the formation of androgens and estrogens but may also be a possible “oocyte factor” to regulate Ca^2+^ channels [27]. The metabolites mentioned above may serve as potential biomarkers for female giant panda mate preference. Increased production of these metabolites correlates with the female’s positive responsiveness toward a specific male, accompanied with physiological changes including estrus initiation or ovulation promotion. It should be emphasized that these findings are solely derived from environmental biomarker metabolomic analysis, and the functional roles of these metabolites require further validation through targeted individual experiments.

We performed KEGG pathway enrichment analysis of differential metabolites, and the results showed that steroid hormone biosynthesis, phenylalanine metabolism, and tropane, piperidine, and pyridine alkaloid biosynthesis were significantly enriched. In the previous study, steroid hormones have been shown to be associated with estrus and ovarian status in giant pandas [8,28]. In other wildlife studies, steroid hormones and their metabolites in feces or urine are an effective means of monitoring reproductive status [29,30]. Phenylalanine is an essential amino acid in the body of animals, which is crucial for the maintenance of life activities [31]. Furthermore, phenylalanine is associated with the production of dopamine and adrenaline [32]. As a result, we speculate that the enrichment of the phenylalanine metabolic pathway suggests a potential upregulation in the anabolic metabolism of dopamine and adrenaline in the preference group. The third enriched pathway was tropane, piperidine, and pyridine alkaloid biosynthesis, which we speculated was related to metabolites in the food ingested by giant pandas. These metabolic pathways associated with food-derived metabolites may not be directly associated with mate preference.

However, this study has several limitations. First, due to the particularity of the giant panda species, it was difficult to include a large number of individuals in this trial, which may compromise the statistical results and the generalizability of the conclusions. This also represents a primary reason why our differential metabolite data could not pass the FDR multiple-testing correction. Second, the differential metabolites identified in the anal gland secretions between the two groups of giant pandas screened in this study lack further experimental validation to determine whether these metabolites are unique to anal gland secretions or also exist in other bodily fluids. Additionally, inherent differences among female individuals, the degree of acceptance of male individuals by females, and environmental factors during sampling may have potentially influenced the trial results. Although these findings are currently hypothetical, they still provide a direction for researching giant panda mate preferences and enhancing natural reproduction rates.

## 5. Conclusions

In conclusion, we identified potential physiological markers from environmental biomarkers that may serve as indicators for female giant panda mate preference. Collectively, these results suggest several potential physiological indicators associated with mate preference, such as prostaglandin G2, prostaglandin B2, palmitoylcarnitine, and estrone, although further validation is needed. Pathway enrichment analysis further revealed novel physiological signatures associated with mate preference, providing new insights into underlying metabolic adaptations. These results advance our understanding of physiological changes in giant panda reproduction and may inform refined conservation strategies.

## Data Availability

The original contributions presented in this study are included in the article and Supplementary Material. Further inquiries can be directed to the corresponding author.

## Supplementary Materials

The following supporting information can be downloaded at: https://www.mdpi.com/article/10.3390/ani15192873/s1. Table S1. Differential metabolites between the preferred and non-preferred groups.
